# A preliminary investigation of precise visualization, localization, and resection of pelvic lymph nodes in bladder cancer by using indocyanine green fluorescence-guided approach through intracutaneous dye injection into the lower limbs and perineum

**DOI:** 10.3389/fonc.2024.1384268

**Published:** 2024-05-22

**Authors:** Yunmeng Zhang, Xinyu Guo, Yueying Zhang, Jinzheng Wei, Pengyu Yan, Haiming Kang, Yang Shu, Chao Liu, Xiaofeng Yang

**Affiliations:** ^1^ Department of Urology, First Hospital of Shanxi Medical University, Taiyuan, China; ^2^ The First Clinical Medical College of Shanxi Medical University, Taiyuan, China; ^3^ Public Experiment Center, University of Shanghai for Science and Technology, Shanghai, China; ^4^ The Second Clinical Medical College of Shanxi Medical University, Taiyuan, China

**Keywords:** bladder cancer, pelvic lymph node dissection, near-infrared fluorescence imaging, indocyanine green, precise visualization

## Abstract

**Objective:**

This study aimed to investigate the feasibility and effectiveness of using indocyanine green (ICG) injected intracutaneously through the lower limbs and perineum for visualized tracking, localization, and qualitative assessment of pelvic lymph nodes (LNs) in bladder cancer to achieve their accurate resection.

**Methods:**

First, ICG was injected into the LN metastasis model mice lower limbs, and real-time and dynamic *in vivo* and ex vivo imaging was conducted by using a near-infrared fluorescence imaging system. Additionally, 26 patients with bladder cancer were enrolled and divided into intracutaneous group and transurethral group. A near-infrared fluorescence imaging device with internal and external imaging probes was used to perform real-time tracking, localization, and resection of the pelvic LNs.

**Results:**

The mice normal LNs and the metastatic LNs exhibited fluorescence. The metastatic LNs showed a significantly higher signal-to-background ratio than the normal LNs (3.9 ± 0.2 vs. 2.0 ± 0.1, p < 0.05). In the intracutaneous group, the accuracy rate of fluorescent-labeled LNs was 97.6%, with an average of 11.3 ± 2.4 LNs resected per patient. Six positive LNs were detected in three patients (18.8%). In the transurethral group, the accuracy rate of fluorescent-labeled LNs was 84.4%, with an average of 8.6 ± 2.3 LNs resected per patient. Two positive LNs were detected in one patient (12.5%).

**Conclusion:**

Following the intracutaneous injection of ICG into the lower limbs and perineum, the dye accumulates in pelvic LNs through lymphatic reflux. By using near-infrared fluorescence laparoscopic fusion imaging, physicians can perform real-time tracking, localization, and precise resection of pelvic LNs.

## 1 Introduction

Bladder cancer is one of the top 10 most commonly occurring cancers worldwide. In 2020, there were approximately 573,000 new cases globally, with over three-quarters of the cases occurring in males (1). The American Cancer Society estimates that in 2023, there will be over 82,000 new cases and over 16,000 deaths from bladder cancer in the United States; these numbers indicate a significant burden on human health (2). Radical cystectomy (RC) is an important surgical approach for treating muscle-invasive bladder cancer (MIBC). However, the prognosis of patients undergoing RC alone is poor, with a 5-year survival rate of only 42% (3). This is primarily due to the high propensity of MIBC to metastasize to the lymph node (LN). Approximately 25% of MIBC patients already have LN metastasis at the time of surgical treatment (4), and patients with metastasis have a relatively poor prognosis (5). Therefore, RC combined with pelvic LN dissection (PLND) is considered the gold standard for treating MIBC.

PLND is a surgical procedure that involves the removal of lymphatic tissue surrounding the blood vessels within a specific range in the pelvic region. It has diagnostic and therapeutic significance in surgical procedures for bladder cancer (6), prostate cancer (7), and colorectal cancer (8). RC combined with PLND can improve patient survival outcomes, even in patients with negative LNs. This is because PLND allows for more accurate LN staging and may remove micrometastases, thereby reducing tumor burden (9, 10).

In the past few decades, the concept and diagnostic approach of sentinel lymph node biopsy (SLNB) have gained significant attention in bladder cancer. Morton (11) first introduced the concept of sentinel lymph node (SLN) in 2004 to describe metastatic LN. The SLN is the first regional LN that directly receives lymphatic drainage from the primary tumor site. In theory, by detecting the SLN, it is possible to determine whether metastasis has occurred in all LNs draining from the cancer site, thus enabling to determine the extent of LN dissection.

In recent years, the use of near-infrared (NIR) fluorescence imaging-guided SLN detection has become a real-time and visually accessible technique. Indocyanine green (ICG) is an FDA-approved NIR fluorescent contrast agent that has been used in the medical field for over 60 years. Compared to the visible light range (400–700 nm), both the excitation light (780 nm) and emission light (820 nm) of ICG are within the NIR range (700–900nm), thereby reducing tissue scattering and autofluorescence interference and increasing imaging depth. Therefore, ICG has potential advantages for use in imaging (12). ICG-guided SLN detection has been successfully applied in clinical practice for the diagnosis of breast cancer (13), penile cancer (14), and melanoma (15). However, the complexity of bilateral lymphatic drainage in the bladder leads to a high false-negative rate and low detection rate, which limits the application of ICG in bladder cancer SLNB (16, 17).

In a preliminary study, Rietbergen et al. (18) demonstrated the feasibility of the hybrid tracer ICG-^99m^Tc-nanocolloid for SLNB in clinical node-negative MIBC patients scheduled for RC with extended PLND (ePLND). However, its study was limited by sample size and lack of outcome data. In another study (19), ICG fluorescence-guided dissection of sentinel regions was able to visualize the lymphatic drainage pathways of the tumors in all RC patients.

The main routes of ICG injection include intravenous injection, intradermal injection, submucosal injection, and intracavitary injection (20). Currently, the reported injection sites for ICG tracing of pelvic LNs in bladder cancer include submucosal or detrusor muscle around the tumors. However, factors such as complex operation techniques and unstable imaging effects have led to a low success rate of visualization (21).

Thompson et al. (22) first proposed the concept of indirect lymphangiography in 1966, which is safer and simpler than direct lymphangiography. The principle of this method involves intracutaneous or subcutaneous injection of the contrast agent, which then reaches the lymphatic vessels through tissue spaces and is subsequently drained into the LNs, thereby allowing for local visualization of the LNs and lymphatic vessels by X-ray imaging. Shinaoka et al. (23) established a new lymphatic imaging protocol by performing detailed anatomical analysis of the LNs and lymphatic vessels through subcutaneous injection of ICG into the lower limbs. Therefore, based on the successful development of the Saians® 4K fluorescence imaging medical device, our research group combined indirect lymphangiography and NIR fluorescence imaging technology to design a novel approach by using intracutaneous injection of ICG through the lower limbs and perineum for labeling pelvic LNs in bladder cancer. This approach aims to provide real-time visualization guidance for accurate resection during surgery. We also performed intravesical injection of ICG around the bladder tumor through the urethra to differentiate the two injection methods on the basis of localization effects on pelvic LNs and positive LNs. This further validates the effectiveness and feasibility of the intracutaneous injection of ICG through the lower limbs and perineum.

## 2 Materials and methods

### 2.1 Animal model

#### 2.1.1 Cell line

The present study used the green fluorescent protein (GFP)-labeled 5637 bladder cancer cell line (5637-GFP). The cell line was cultured in RPMI-1640 medium supplemented with 10% fetal bovine serum and 1% antibiotics. The cells were incubated at 37°C in a humidified atmosphere containing 5% CO_2_.

#### 2.1.2 Establishment of animal models and NIR fluorescence imaging

All experimental procedures, including animal handling and care, were conducted in accordance with the ethical guidelines of the Ethics Committee for Animal Welfare of Shanxi Medical University. Twelve female BALB/C-nu mice (age: 4–6 weeks, weight: 12–16 g, obtained from Beijing Huafukang Bio-technology Co.,China) were randomly assigned to a control group (n = 6) and a footpad tumor LN metastasis experimental group (n = 6). In the experimental group, 5637-GFP cells (4 × 10^6^) were injected into the right footpad of mice. After 4 weeks, a footpad tumor metastasis model was established. The control group received no treatment or intervention.

ICG (25 mg, Dandong Medical Creation Pharmaceutical Co., China) was prepared as a 0.25 mg/mL solution. Forty microliters of the solution was intracutaneously injected into the right footpad of both experimental and control group mice. Real-time dynamic fluorescence imaging was performed to observe the distribution of ICG in mice. After 6 h, the fluorescent-labeled tissues were excised under fluorescence guidance. The fluorescence intensity of the labeled tissues and the surrounding tissues was analyzed using ImageJ software, and the signal-to-background ratio (SBR) was calculated. The tissues were then prepared as frozen sections. Under a fluorescence microscope, the GFP signal was detected at excitation and emission wavelengths of 480 and 520 nm, respectively, while the ICG signal was detected at excitation and emission wavelengths of 780 and 820 nm, respectively. Finally, tissue pathological analysis was performed by hematoxylin-eosin staining.

### 2.2 Patients and methods

#### 2.2.1 Study population

This study was approved by the Ethics Committee of the First Hospital of Shanxi Medical University and registered with the Chinese Clinical Trial Registry. From June 2022 to December 2022, based on imaging examinations and cystoscopy, and after excluding contraindications, we included a total of 26 patients who were diagnosed with MIBC before surgery and planned to undergo RC+PLND. On another hand, the exclusion criteria are also considered that including the patients with a history of pelvic surgery or the tumors outside the bladder. The patients were divided into the lower limbs and perineum intracutaneous injection group (intracutaneous group) and transurethral intravesical injection group (transurethral group) according to the randomization principle. Finally, 16 patients were included in the intracutaneous group and 10 patients were included in the transurethral group.

#### 2.2.2 Imaging system equipment

**Figure 1:**
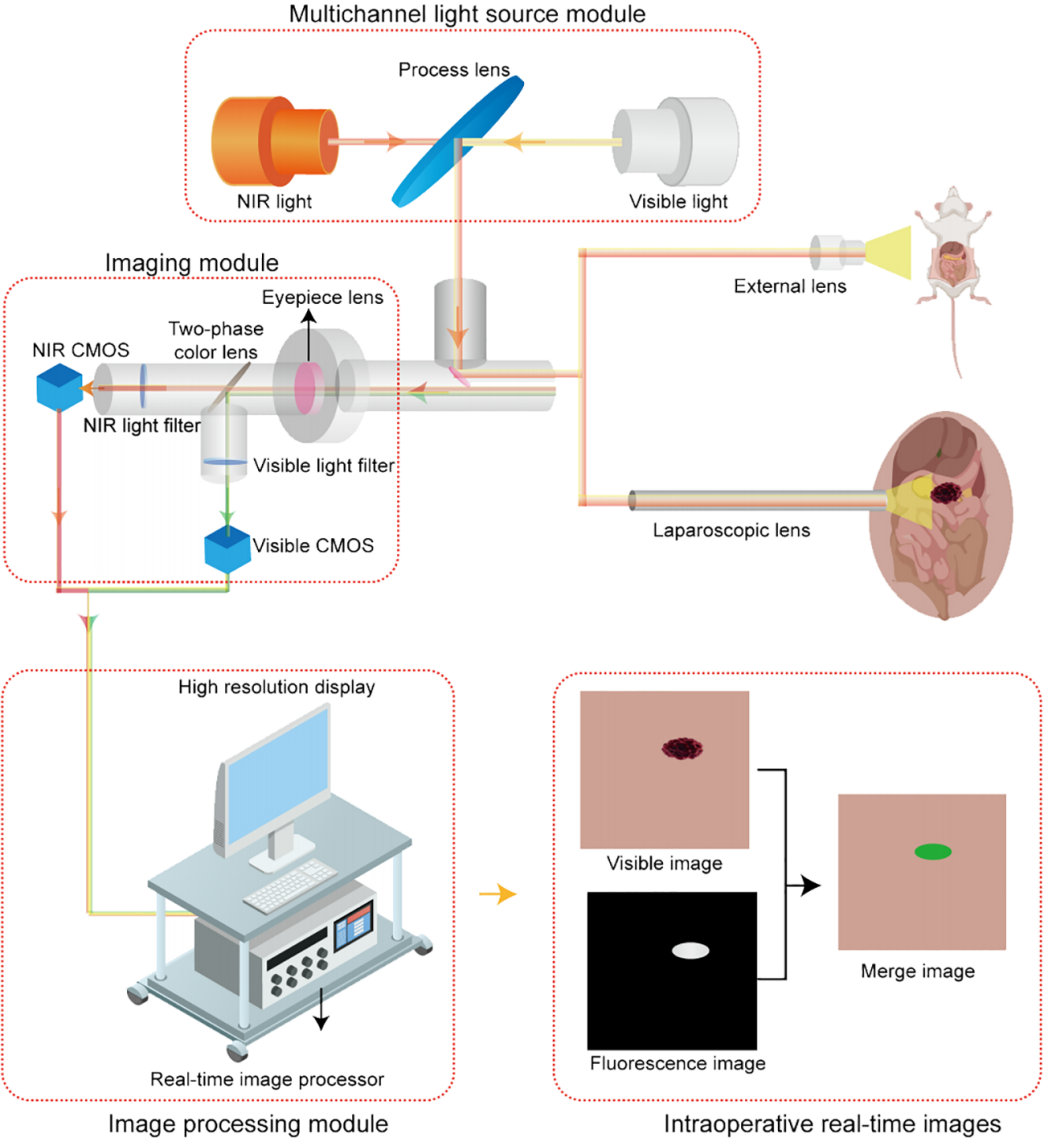
Schematic diagram of the imaging principle of the Saians® 4K fluorescence system.

The Saians® 4K fluorescence imaging system (Saians Technology Development Co., Ltd., Taiyuan, Shanxi, China) is equipped with an ex vivo imaging lens and a laparoscopic lens for *in vivo* imaging. Both lenses offer visible light, fluorescence, and fusion pseudo-color modes. The visible light mode provides clear anatomical images, the fluorescence mode displays white fluorescence images, and the fusion mode simultaneously displays both anatomical and fluorescence images in a pixel-level fused green pseudo-color image within the same surgical field. These three modes do not require switching during the operation, which enables to achieve real-time intraoperative visualization. The imaging schematic is shown in **Figure 1**.

#### 2.2.3 Methods of ICG injection

ICG was diluted to 2.5 mg/ml concentration by using sterile injectable water. For the intracutaneous group, at 1.5 h before the surgery, 0.5 ml ICG was injected intracutaneously at a point 2 cm above the medial malleolus on each side and on both sides of the midline of the perineum; the total volume was 2 ml. Immediately after the injection, each injection site was gently massaged. The ex vivo imaging lens of the imaging system was used to observe the fluorescence image of ICG traveling upward along the injection sites, and the time to reach the inguinal LNs was recorded. For the transurethral group, at 1.5 h before the surgery, 2 ml of ICG was injected into the submucosa around the bladder tumor by using a bladder injection needle, and the bladder was then filled with normal saline for 30 min to provide moderate bladder distension, thereby promoting lymphatic drainage.

#### 2.2.4 Methods of fluorescence-guided PLND

The surgeries in both groups were performed by the same team. The patients were induced with general anesthesia and placed in the supine position with their head down and their feet elevated. A fluorescence laparoscope was inserted using the conventional laparoscopic approach. The procedure consisted of two steps: fluorescence-guided PLND (F-PLND) followed by RC. During F-PLND, a fluorescence-guided localization and excision technique was used. First, the position of the fluorescence image was observed before the peritoneum was incised. The peritoneum was then opened along the external iliac vessels, thereby exposing the external iliac arteries and veins, internal iliac arteries and veins, common iliac vessels, and obturator nerve. The tissue marked with fluorescence was then separated along the edge observed in the fluorescence image, excised, and subsequently numbered and sent for histopathological examination.

### 2.3 Statistical analysis

All graphs, calculations, and data analysis were performed using GraphPad Prism software. For quantitative data conforming to a normal distribution, we represented them using mean ± standard deviation and two independent-sample t-test was used to analyze the difference in SBR and the difference in the number of LN detected between the two groups. For qualitative data, we represented them using probabilities and percentages, and chi-square test (χ2-test) and Fisher probability method was used to analyze the difference in LN accuracy rate and the difference in LN positive detection rate between the two groups. P-values were considered statistically significant at P < 0.05.

## 3 Results

### 3.1 Fluorescence imaging characteristics of ICG injected in the lower limbs in normal and LN metastasis mice

**Figure 2:**
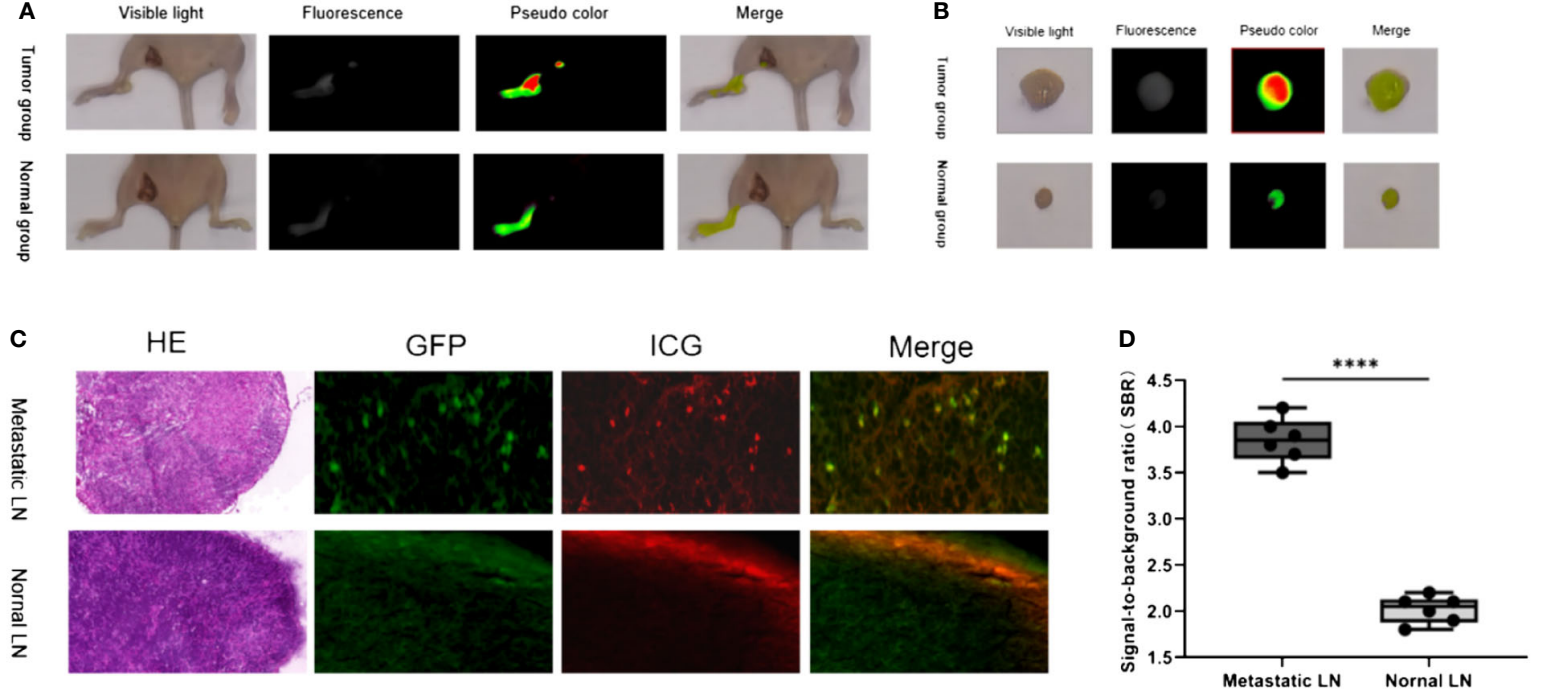
Fluorescence imaging characteristics of ICG injected in the lower limbs in normal and LN metastasis mice. **(A)** *In vivo* imaging of LN in tumor group and normal group; **(B)** *In vitro* imaging of LN in tumor group and normal group; **(C)** Representative H&E staining, GFP, ICG, and merged microscopic images of normal LNs and metastat LNs; **(D)** The SBR of metastatic and normal LNs. ****: P< 0.0001.

The LN metastasis mouse model was successfully established by injecting tumor cells through the footpad, as confirmed by the GFP signal in the tissues and pathological examination (**Figure 2**). After 6h of ICG injection in the lower limbs, fluorescence imaging was observed in both the control group with normal LNs and the experimental group with metastatic LNs. However, the experimental group showed significantly higher SBR than the control group (3.9 ± 0.2 vs. 2.0 ± 0.1). The t-test was used to analyze the difference in SBR between the two groups of mice, and the results were statistically significant (p<0.05). Microscopic imaging revealed that the fluorescence imaging of ICG in the metastatic LNs overlapped partially with the GFP-labeled tumor cells, thus indicating a higher accumulation of ICG in the LNs with tumor cell metastasis.

### 3.2 ICG-guided fluorescence imaging characteristics of pelvic LN resection in patients with bladder cancer

The surgeries in both groups of patients were successfully completed. No intraoperative or postoperative adverse reactions related to ICG injection were observed in any of the patients. In the intracutaneous group, successful visualization of pelvic LN was achieved in all 16 patients. In the transurethral group, successful visualization of pelvic LN was achieved in 8 of 10 patients, and the remaining 2 patients were not included in the statistical analysis. General patient information is provided in **Table 1**.

**Table 1:** General information of the patients.

Patient information	intracutaneous group (n=16)	transurethral group (n=8)	P
Sex (number)			>0.05
Male	12	5	
Female	4	3	
Age (years)			>0.05
Average	68.1±8.6	67.3±9.6	
Range	58-87	50-79	
pT stage (number)			> 0.05
pT2	9	5	
pT3	6	2	
pT4	1	1	
pN stage (number)			>0.05
pNO	13	7	
pN1	2	0	
pN2	1	1	

#### 3.2.1 Imaging findings of the intracutaneous group

**Figure 3:**
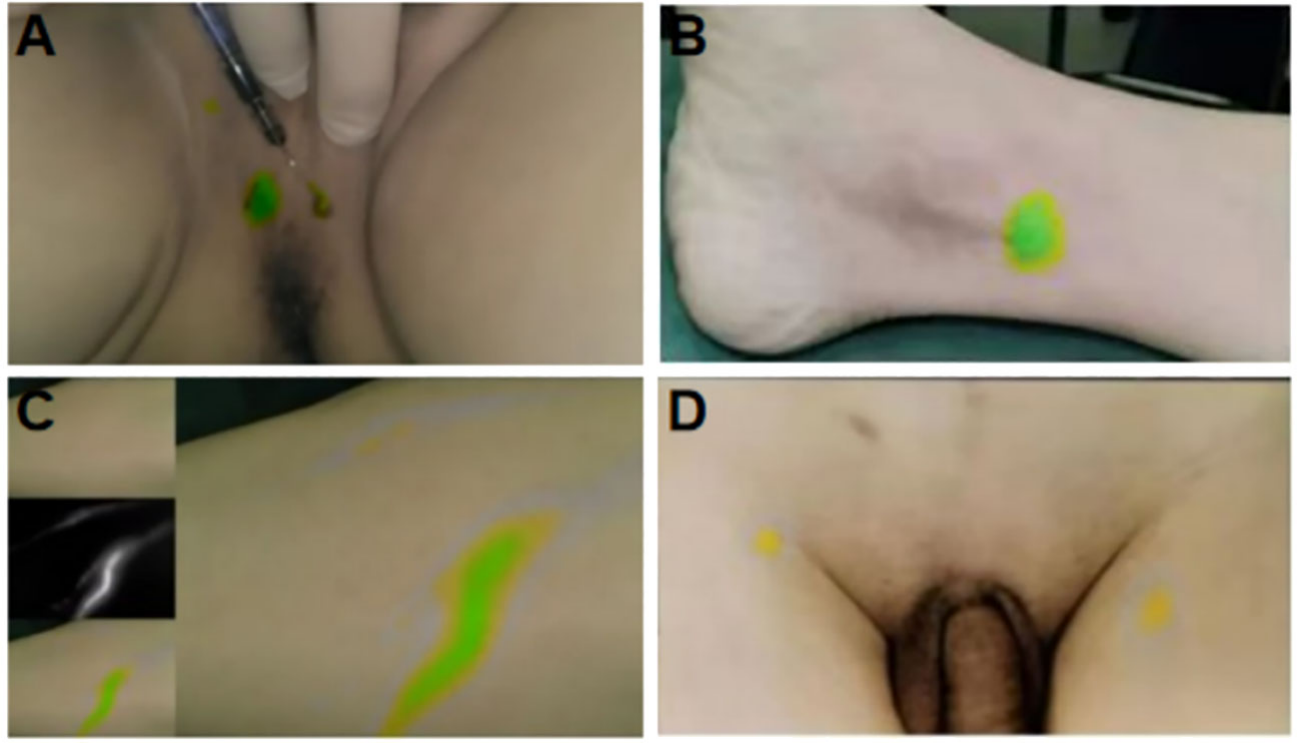
Fluorescent imaging of ICG injection site and its flow in lower limb LNs. **(A)** The injection site of lower limbs; **(B)** The injection site of perineum; **(C)** Fluorescent image of lymph flowing in lymphatic vessels after ICG lower limb injection; **(D)** Fluorescent imaging of inguinal LNs.

**Figure 4:**
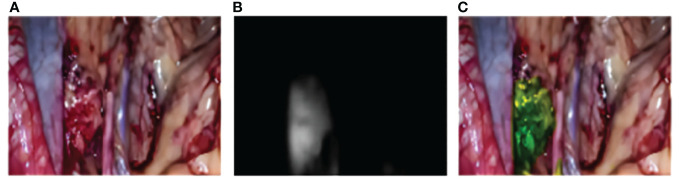
Intraoperative multimodal fluorescence imaging of obturator LN. **(A)** The visible light model; **(B)** The fluorescence model; **(C)** The merge model.

**Figure 5:**
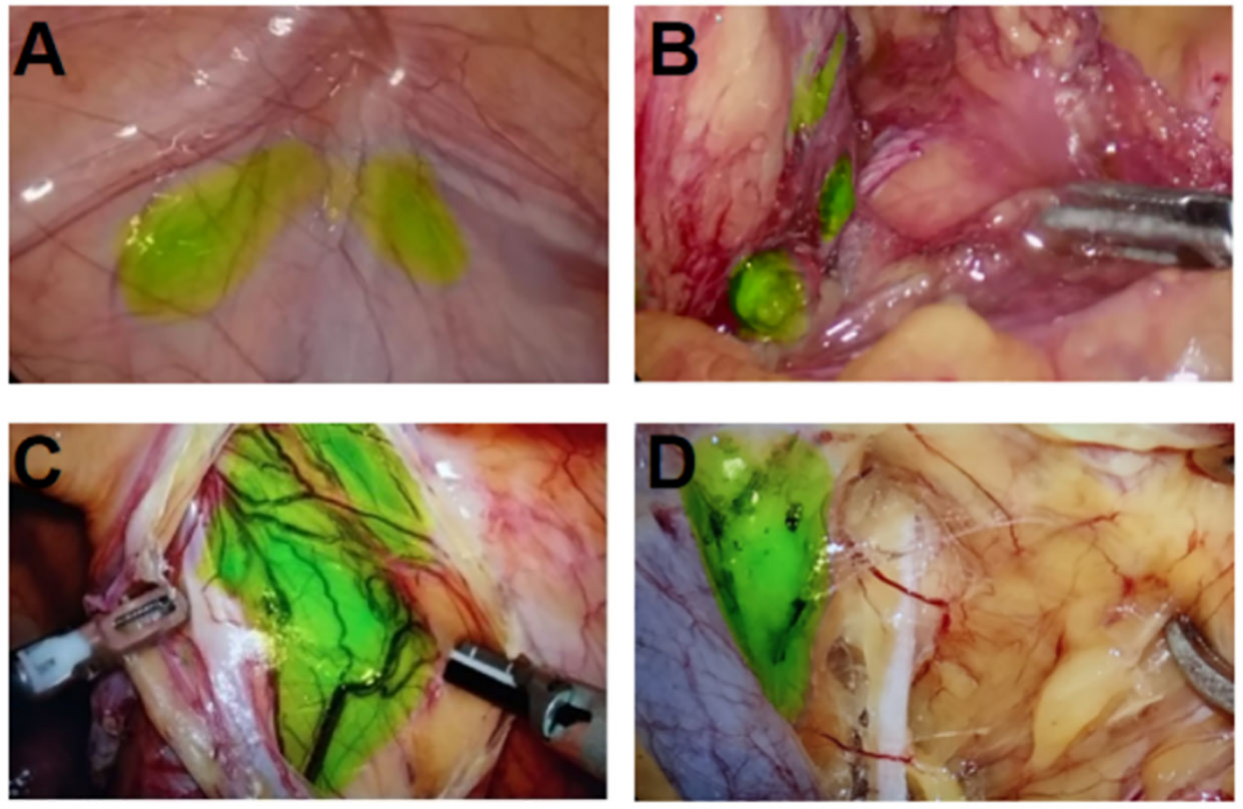
Resection of pelvic LNs in merge mode after ICG injection in the lower limbs combined with perineum. **(A)** Intra-abdominal iliac artery LN; **(B)** interiliac vessel LN; **(C)** Cloquet LN next to the circumflex iliac vein; **(D)** The obturator LN between the external iliac vein and the obturator nerve.

Following ICG injection, the external lens of the imaging system was initially focused on the injection site. A fluorescence image of the lower limbs was first acquired, and the system gradually moved toward the proximal end of the limbs. The system passed through the inner side of the calf, popliteal fossa, and inner side of the thigh, finally reaching the inguinal region. The time taken to reach the inguinal LN was 25–35 min (**Figure 3**). Before the peritoneum was opened intraoperatively, fluorescence imaging was performed at the distal and proximal ends of the iliac vessels and their surrounding areas. After opening the peritoneum along the external iliac vessels, circular, elliptical, and linear green fluorescence images were observed around the internal iliac vein, adjacent to the internal iliac artery, and near the iliolumbar vessel (**Figure 4**). We found that the fusion pseudo-color mode accurately displayed the LNs and lymphatic vessels, with a clear boundary from the surrounding nerves and blood vessels (**Figure 5**). Intracutaneous ICG injection into the lower limbs and perineum allowed visualization of the external iliac artery, internal iliac artery, common iliac vessels, and the obturator LN. The surgeons performed LN dissection using the fusion mode, and the LNs were numbered and sent for histopathological examination. 82 fluorescent-labeled tissues were removed from the 16 patients during the surgery. Postoperative pathological results showed 80 LN tissues and 2 fibroconnective tissues, with an accuracy rate of 97.6% for the fluorescent-labeled LN tissues. A total of 180 LNs were detected in the fluorescent-labeled tissues, with an average of 11.3 ± 2.4 LNs removed per patient. Three of these patients (18.8%) had a total of 6 positive LNs.

#### 3.2.2 Imaging findings of the transurethral group

Among the 10 patients in the transurethral group, one patient experienced extensive leakage of ICG outside the bladder wall and peritoneum, resulting in blurred fluorescence imaging of the LNs and inability to identify them. One patient did not undergo bladder perfusion, resulting in nonvisualization of the LNs. In the remaining 8 patients, 32 fluorescent-labeled tissues were removed. Postoperative pathological results showed 27 LN tissues and 5 fibroconnective tissues, with an accuracy rate of 84.4% for the fluorescent-labeled LN tissues. A total of 69 LNs were detected in the fluorescent-labeled tissues, with an average of 8.6 ± 2.3 LNs removed per patient. Among these, 2 positive LNs were detected in one patient (12.5%).

**Figure 6:**
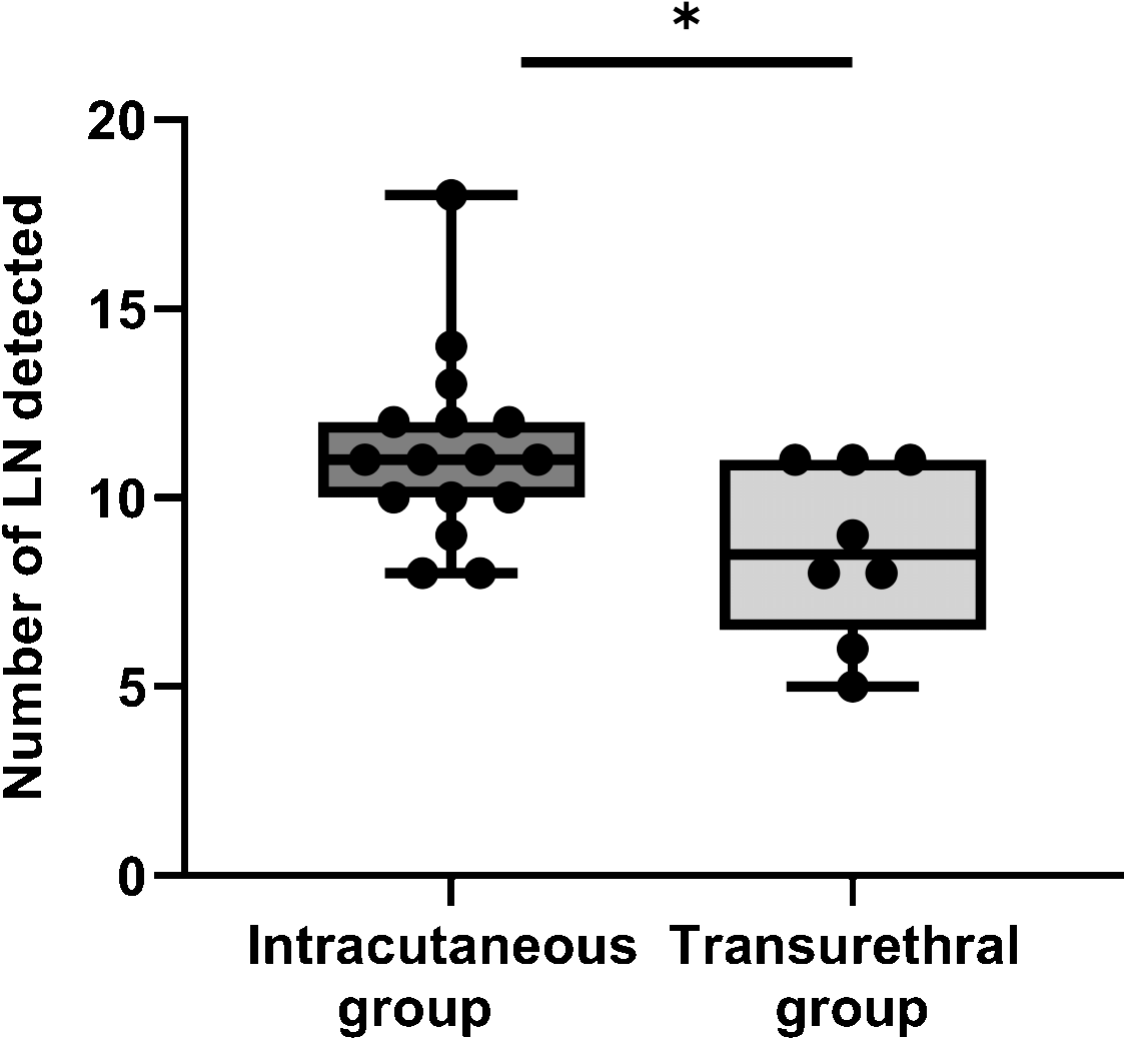
The number of LN detected between intracutaneous group and transurethral group. *: P< 0.05.

The data results show that: the accuracy rate and detection number of fluorescent-labeled LN tissues were significantly higher in the intracutaneous group than in the transurethral group, and the results were statistically significant (p < 0.05). However, no significant difference was observed in the positive detection rate of LN between the two groups (p > 0.05). The results are shown in **Figure 6**.

## 4 Discussion

Pelvic LNs are the primary route of metastasis in bladder cancer and an important indicator of patient prognosis (24). RC combined with PLND is the standard surgical approach for treating MIBC. However, the precise localization of pelvic LNs during the surgery remains a substantial challenge. F-PLND utilizes NIR fluorescence imaging to provide surgeons with high SBR for differentiating LN from the surrounding tissues, thereby effectively avoiding damage to the surrounding tissues and improving the efficiency of PLND.

The distribution of pelvic LN metastasis has always been a hot topic in urological tumors such as bladder cancer and prostate cancer (25). Gandaglia and Gondoputro evaluated the safety and effectiveness of ^99m^Tc-based prostate-specific membrane antigen (PSMA) in robot-assisted radioguided surgery, and considered that this technology is valuable in detecting LN metastasis. However, its sensitivity is not high and therefore cannot replace conventional ePLND (26, 27). Francesco et al. correctly staged 98% of prostate cancer patients using ICG-guided lymphography, and the negative predictive value as high as 97% indicates that avoiding ePLND is safe for most patients (28). Similarly, another reported study evaluated conventional ePLND and PLND guided by ICG, it was found that adding fluorescence can enhance the identification rate of lymphatic drainage, and obtain more LNs and metastatic LNs, providing a more accurate local staging and prolonging recurrence-free survival (29).

In 2007, Knapp et al. (30) conducted the first study on intravesical injection of ICG for SLN mapping in an animal model. The authors emphasized that the control of intravesical pressure is a crucial factor to achieve the optimal imaging of SLNs and that the tracking of SLNs must be individualized because of anatomical variations in lymphatic drainage. In 2014, Schaafsma et al. (31) proposed that the intravesical injection of ICG with bladder distention is the optimal method to visualize SLNs. Subsequently, Manny et al. (32) identified SLNs in bladder cancer patients by using robot-assisted ICG fluorescence laparoscopy and reported 75% sensitivity and 52% specificity for positive LNs. However, Liedberg et al. (33) indicated that SLN detection in bladder cancer had a false negative rate of up to 19%.

The above-mentioned findings indicate that SLN detection in tumors of the urinary system, such as bladder cancer and prostate cancer, still lacks reliable theoretical basis (34). Transurethral intravesical injection of ICG not only carries the risk of urethral injury and infection but also poses challenges in controlling the injection site, dosage, and depth, leading to potential issues such as ICG leakage and intraoperative fluorescence contamination. The thickness of the bladder wall, intravesical pressure, and degree of bladder distention can also affect the stability of intraoperative ICG imaging. In the transurethral group, two patients did not show LN visualization intraoperatively because of inadequate control of parameters such as injection depth and intravesical pressure. Other studies have also confirmed that SLN mapping should be considered a supplementary approach to PLND during RC, rather than as a replacement of routine bilateral PLND (17).

The real benefits of ICG are represented by the radiation free procedure, the easy access and visualization of the targeted LNs as lymphatic route. However, although SLN tracing based on transurethral intravesical injection of ICG has certain clinical application value, it also has limitations such as high false negative rate, non-standardized procedures and a range of imaging influencing factors, which require further improvement. Additionally, due to the limitation of penetration depth, ICG may affect target observation in obese patients (35). Therefore, we believe that the current role of ICG in RC+PLND is to provide targeted and accurate anatomical guidance rather than being used for SLN applications.

Although the three-tier template for PLND was proposed as early as 2004 by Leissner et al. (36), there is still no consensus on the optimal extent of PLND. Bruins et al. (37)found that any type of PLND template was superior to no PLND. A larger dissection range may remove more LNs and potentially provide better survival benefits. However, it can also lead to various complications such as lymphatic cysts, obturator nerve injury, and lymphedema (38). Therefore, the determination of the ideal dissection range for implementing more precise PLND is highly clinically relevant.

In the present study, we analyzed the lymphatic drainage system of the normal lower limbs, perineum, and bladder (39, 40). We found that the LNs draining the normal pelvic organs and those associated with tumor metastasis are the intrinsic LNs of the pelvic anatomy, including the internal and external iliac, obturator, and presacral LNs. A previous study (41) reported that the use of ICG and iodine injections into the lower limbs to determine the lymphatic reflux pathway provides a theoretical basis for our experiment. Therefore, we combined indirect lymphangiography techniques with NIR fluorescence imaging to investigate the feasibility of locating and identifying pelvic LNs through ICG injection into the lower limbs and perineum.

First, we established a model of LN metastasis in mice and analyzed LN imaging after ICG injection in the footpad. We found that this injection method was feasible, and the LNs in both control and experimental groups showed successful imaging, with higher SBR in the experimental group than in the control group. Based on these results, we suggest that both normal and metastatic LNs can be identified in mice.

A comparison with the classical approach of transurethral intravesical injection of ICG showed that ICG injection in the lower limbs and perineum can detect more LNs, with an accuracy rate of 97.3% for LN identification. This may be related to the fact that lymphatic vessels in fibroconnective tissues are fluorescently labeled and removed together. Additionally, we found that intraoperative damage to small lymphatic vessels or LNs may cause leakage of ICG, leading to fluorescence contamination in the local surgical field, thus affecting the localization and removal of LNs. Therefore, surgeons should try to be gentle during the operation and avoid excessive separation of large tissue. Both groups showed positive fluorescence imaging of LNs during the surgery, although the difference was not statistically significant. Inoue et al. (42) first reported that the intravesical injection of ICG could visualize metastatic LNs in patients with bladder cancer. The results showed a false-negative rate of 100%, and it was suggested that when the lymphatic pathway is completely blocked by tumor cells, an alternative pathway may be induced. ICG, as a nontargeted contrast agent, may be more suitable for detecting pelvic negative LNs rather than metastatic LNs. However, in another study (43), the intravenous injection of ICG successfully visualized metastatic LNs in mice and tumor patients with an accuracy rate of 83.3%. This finding holds promise as a new method for identifying LN metastasis.

The present study confirmed that ICG injection in the lower limbs and perineum can assist surgeons in the precise localization and removal of LNs during F-PLND, thereby minimizing unnecessary extensive LN dissection and reducing intraoperative damage. Considering the importance of LN dissection in the prognosis of bladder cancer, the localization and tracking of F-PLND should be a focus of future research studies (44).

Although we successfully visualized pelvic LNs by injecting ICG in the lower limbs and perineum, thereby effectively improving the accuracy of LN identification and the efficiency of LN dissection during surgery, the present study has some limitations. Firstly, we did not remove fluorescence-negative tissues to analyze the specificity of this injection method for visualizing pelvic LNs. This will be a direction of our further research. Secondly, this technique cannot evaluate the status of LNs intraoperatively. In the future, interdisciplinary research across multiple fields will be needed to exploit targeted probes to address this issue. Lastly, this is a single-center small sample study, and the results need further validation in multicenter studies with larger samples.

## 5 Conclusions

This study confirms that after intradermal injection of ICG in the lower limbs and perineum, ICG can accumulate in the pelvic LNs through lymphatic reflux. Compared to intravesical injection, the operation method is simpler, safer and the intraoperative imaging effect is more stable. Under the guidance of F-PLND, surgeons can real-time track, accurately locate and precisely remove pelvic LNs, greatly improving the efficiency of PLND. This technique will provide a new theory and method for further research on the accurate localization and qualitative analysis of pelvic LNs during surgery.

## Data availability statement

The original contributions presented in the study are included in the article/supplementary material. Further inquiries can be directed to the corresponding author.

## Ethics statement

The studies involving humans were approved by the ethics committee of the First Hospital of Shanxi Medical University. The studies were conducted in accordance with the local legislation and institutional requirements. The participants provided their written informed consent to participate in this study. The animal study was approved by the Ethics Committee for Animal Welfare of Shanxi Medical University. The study was conducted in accordance with the local legislation and institutional requirements. Written informed consent was obtained from the individual(s) for the publication of any potentially identifiable images or data included in this article.

## Author contributions

YnZ: Writing – original draft, Writing – review & editing. XG: Writing – original draft, Writing – review & editing. YeZ: Writing – original draft, Writing – review & editing. JW: Writing – review & editing. PY: Writing – review & editing. HK: Writing – review & editing. YS: Writing – review & editing. CL: Writing – original draft, Writing – review & editing. XY: Writing – original draft, Writing – review & editing.
